# Owners’ Perceptions of Their Animal’s Behavioural Response to the Loss of an Animal Companion

**DOI:** 10.3390/ani6110068

**Published:** 2016-11-03

**Authors:** Jessica K. Walker, Natalie K. Waran, Clive J. C. Phillips

**Affiliations:** 1 New Zealand Companion Animal Council, P.O. Box 43221, Auckland 2351, New Zealand; 2 Faculty of Education, Humanities and Health Science, Eastern Institute of Technology, Napier 4112, New Zealand; nwaran@eit.ac.nz; 3 Centre for Animal Welfare and Ethics, School of Veterinary Sciences, University of Queensland, Gatton, QLD 4343, Australia; cphillips@uq.edu.au

**Keywords:** animal-animal bond, animal grief, cat, companion animal bond, dog

## Abstract

**Simple Summary:**

The loss of a companion animal is recognised as being associated with experiences of grief by the owner, but it is unclear how other animals in the household may be affected by such a loss. This paper investigates the behavioural responses of dogs and cats to the loss of an animal companion through owner-reported observations. There was consensus that behaviour changed as a result of loss including increased affectionate behaviour, territorial behaviour, and changes in food consumption and vocalisation.

**Abstract:**

The loss of a companion animal is recognised as being associated with experiences of grief by the owner, but it is unclear how other animals in the household may be affected by such a loss. Our aim was to investigate companion animals’ behavioural responses to the loss of a companion through owner-report. A questionnaire was distributed via, and advertised within, publications produced by the Royal Society for the Prevention of Cruelty to Animals (RSPCA) across Australia and New Zealand, and through a selection of veterinary clinics within New Zealand. A total of 279 viable surveys were returned pertaining to 159 dogs and 152 cats. The two most common classes of behavioural changes reported for both dogs and cats were affectionate behaviours (74% of dogs and 78% of cats) and territorial behaviours (60% of dogs and 63% of cats). Both dogs and cats were reported to demand more attention from their owners and/or display affiliative behaviour, as well as spend time seeking out the deceased’s favourite spot. Dogs were reported to reduce the volume (35%) and speed (31%) of food consumption and increase the amount of time spent sleeping (34%). Cats were reported to increase the frequency (43%) and volume (32%) of vocalisations following the death of a companion. The median duration of reported behavioural changes in both species was less than 6 months. There was consensus that the behaviour of companion animals changed in response to the loss of an animal companion. These behavioural changes suggest the loss had an impact on the remaining animal.

## 1. Introduction

Across Australia and New Zealand, the companion animal population comprises 36 million companion animals [1]. Internationally, both New Zealand and Australia have some of the highest cat and dog ownership rates with 44% of New Zealand households and 29% of Australian households owning a cat and 28% of New Zealand and 39% of Australian households owning a dog. This is a substantially greater number than the UK where 17% of households own a cat and 24% a dog and lower than the USA where 44% of households own a dog [1]. Owners of companion animals show strong emotional connections to their pets [2,3] often considering them part of the family and showing affection, support and comfort in similar levels to that of a human family member [4,5,6]. Indeed, in some instances attachment to an animal companion may transcend relationships with other humans [5,7]. Inevitability, due to the vast number of companion animals within today’s society, a large number of owners will experience separation from, or loss of, their companion animal and unsurprisingly such a loss is distressing, often causing the person significant grief and mourning [6,8,9]. The impact of the loss of a companion animal is well documented to result in an experience of grief that is not dissimilar to what one would experience as a result of the loss of a human to which they had a similar level of attachment [7,10,11]. Conversely, research investigating the impact companion animal loss has on any remaining animals within the household is not well documented. 

The study of emotions in animals is essential to our understanding of their welfare, the advancement of which can lead to improvement in their quality of life. In recent years a number of methodologies have been developed and applied in an attempt to measure both positive and negative emotions in animals which may be relevant to the study of grief in animals including: neural homologies [12,13], behaviour and physiology assessment [14,15,16]; human judgment of subjective experience [17], and the measurement of cognitive appraisal of ambiguous stimuli which can signify overarching positive and negative emotional states [18,19]. Although the internal experiences of animals cannot be directly accessed, thereby rendering elusive any proof that animals experience a similar emotional experience of grief to humans, the methodologies described above have evidenced similarities in behavioural, physiological and neuroanatomical responses to separation [20,21], suggesting that some animals may have the capacity to experience negative feelings as a result of the loss of a conspecific [22]. From an evolutionary perspective grief may have evolved from a stress reaction in response to separation from companions, thereby acting to maintain group cohesion in social species [23]. 

For the purposes of this paper, grief is considered from a biological perspective as a response to separation, described by a bi-phase model of “active/passive” behavioural responses [24,25]. This biphasic modality is thought to function to increase the likelihood that separated, bonded animals will be reunited and is characterised by increased searching activity and vocalisations in the first phase and decreased activity and withdrawal from the environment (to conserve energy) in the second phase [24]. The response observed in farm animals following artificial weaning could be considered an example of this. In this situation farm animals attempt to reunite following the separation, and behaviours and physiological changes exhibited include: increased vocalisations, altered feeding patterns, increased locomotion, suspension of play, elevated corticosteroids, heart rate, core body temperature, and reduced immunocompetence [26,27,28,29]. Although there is no conclusive scientific evidence, the abrupt and permanent separation of offspring from their mothers in livestock management systems is increasingly being considered to be a significant emotional stressor for both the mother and the offspring [22]. Furthermore, anecdotal accounts of the emotional distress displayed as a result of the severance of mother-offspring bonds have been documented in wild baboons, elephants, chimpanzees and dolphins including; intense vocalisations and interaction with the deceased comprising of touching and carrying the body for prolonged periods of time before abandonment [30,31,32,33]. Field studies of elephants have documented strong vocal and behavioural reactions (including attempts to rouse the deceased) to the death of a family member [30,34] and the attention that elephants give to bones and carcasses is suggested to be consistent with them comprehending and responding to the death of a conspecific [35]. The bi-phase model of “active/passive” behavioural responses to separation has been used to suggest that primates may experience grief in laboratory settings [36,37,38]. Primate mother-infant separation experiments, carried out primarily in the 1960’s, demonstrated that infants (rhesus monkeys [39,40,41]; bonnet macaques [42]; and pig-tailed macaques [36,43,44]) exhibit an initial period, lasting from several hours to several days [45], of distress and agitation. This response is characterised by increased vocalisation, sleeplessness and activity, and followed by a period of reduced vocalisation, locomotory, exploratory and play activity that resembles depression. Further research has documented similarities between brain regions activated during mother-young separation in the guinea pig and those activated during the human experience of grief [21], which suggests that some animals experience emotional distress similar to the human experience of grief as a result of separation. 

Although a great deal is known about the severance of mother-offspring bonds, little is known about the severance of peer bonds in companion animals [46]. Conspecific bonding in cats and dogs has not been extensively studied, but limited research has demonstrated that puppies have preferred play partners by six months of age [47] and are able to recognise their kin up to two years after separation [48], with social isolation of dogs documented to cause chronic stress [49]. Similarly, distress associated with the separation of littermates has been documented in kittens [50], suggesting that companion animals may form strong social bonds with peers. Indeed, some changes in the behaviour of both dogs and cats following the death of an animal companion have been anecdotally reported by companion animal owners [51]. 

While research on the severance of peer bonds in companion animals is sparse, there is a great deal written about owner-orientated separation anxiety. Dogs, suffering from owner-orientated separation anxiety, show functionally analogous behaviours to human infants in maintaining proximity and displaying stress-related behaviours after brief separation from their owner [52] followed by increased and prolonged displays of affection towards their owner upon reunion [53,54]. Separation from a human owner produces strong responses in over-attached dogs [55,56], which typically include destructive behaviour, escape attempts (to reunite with the owner), increased vocalisation and inappropriate elimination [53,54,55,57,58,59,60]. Dogs may also abstain from eating and drinking [61]. Behaviour symptomatic of separation anxiety in cats appears to parallel those seen in dogs, including inappropriate urination and defecation, increased vocalisation, destructiveness and psychogenic grooming [62]. It is possible that inappropriate urination and defecation, increased vocalisations and destructiveness could be considered to parallel the first phase of the bi-phasic definition of grief, whilst psychogenic grooming and abstaining from eating and drinking parallel the second phase.

The long-lasting relationship between owners and companion animals provides a unique source of inquiry regarding animal emotions [63]. Subjective ratings, observations and reports are a standard component of research within human psychology and include observations by clinicians, caregivers and peers as a substitute for self-report when self-report cannot be obtained [64], for example in the measurement of grief in individuals with intellectual disability, or young children [65,66]. In the case of animals, owners’ assessments are the primary source of information regarding companion animal behaviour problems [67,68,69] with companion animal owners being increasingly utilised as a source of information about their animal’s behaviour [63,70,71]. Indeed, owners have been demonstrated to provide meaningful and consistent reports about the behaviour of their animals [71] and recently, owners have been documented as being able to identify overt behavioural indicators of stress (e.g., trembling, crying or excessive barking) indicative of reduced welfare [72]. Consequently, companion animal owners are an ideal resource, in the first instance, to investigate behavioural changes in companion animals (through owner-perceived reporting) after the loss of an animal companion. 

## 2. Materials and Methods 

### 2.1. Questionnaire Design

The questionnaire was divided into two parts. The first section collected demographic data on the companion animal owner (including age range and gender), up to two deceased companion animals (deceased < 5 years) and up to two remaining companion animals. Data obtained on the deceased animal(s) included species, breed, sex, age at death, date of death, cause of death and source of animal(s). Data obtained on the remaining animal(s) included species, breed, sex, age, whether the remaining animal saw the deceased animal’s body and if so, how he/she reacted to it. Where owners reported on more than one deceased or remaining animal they were able to provide observed behavioural changes separately for each animal. Owners were free to include information on all companion animal species, however the paucity of information pertaining to species other than cats and dogs (rabbits, *n* = 3; guinea pigs, *n* = 1; alpacas, *n* = 1, birds, *n* = 9; rats, *n* = 4; horses, *n* = 6; and humans, *n* = 2) meant that only information pertinent to dogs and cats was analysed. Based on owner descriptions of dog breed, both deceased and remaining, dogs were assigned to one of the seven New Zealand Kennel Club breed group categories (www.nzkc.org.nz/dogselect) or recorded as a crossbreed. Cats were categorised as either pedigree or domestic. The age of animals was categorised as juvenile (0–12 months), adult (1–8 years) or senior (8 years+). 

Section two of the questionnaire related to changes observed in the remaining animals’ behaviour. Based on Schultz et al. [51], we utilised the following seven behavioural categories: feeding, sleeping, vocalisation, elimination, aggression, affection and territoriality. Under each of these behaviour categories owners were asked to indicate if, and how, particular behaviours changed in their remaining animal(s) by encircling the most relevant response, e.g., “increased”, “decreased” or “change”, “no change”. Owners were also asked to estimate the duration of time that behaviour changes persisted. Specifically, using the above categories, owners were asked to comment on the amount and speed of food consumption and whether this affected their remaining animals health; the amount of time spent sleeping and whether the animal avoided his/her normal sleeping location; the amount of time spent vocalising and the volume of vocalisation; the frequency and location changes of elimination; aggression levels directed at animals or people in the household; whether the remaining animal(s) demanded more or less affection, displayed affiliative behaviour, which we termed “clingy/needy” or avoided contact, and whether remaining animals sought out or avoided the deceased animal’s favourite spots, spent increased timing hiding or spent increased time “seeking higher ground”.

### 2.2. Questionnaire Distribution

In New Zealand the questionnaire was distributed to 5500 potential respondents via the SPCA Auckland’s “Animals Voice” magazine (Autumn issue 2010) and through 100 veterinary clinics throughout New Zealand, randomly selected from the veterinary practices listed on the New Zealand Veterinary Association Website (www.nzva.org.nz). Each copy of the aforementioned magazine included an inserted copy of the questionnaire. Each veterinary clinic was sent a research information sheet and a letter requesting that they advertise the questionnaire to their clients, along with 10 copies of the questionnaire. Seventeen clinics declined to participate. In order to ensure respondent anonymity, all questionnaires were returned to a postage paid Private Bag postal address. 

In Australia advertisements for the survey were published in the RSPCA New South Wales quarterly magazine “Animals” (Summer issue 2010/2011) distributed to 6800 readers, the RSPCA Queensland’s online newsletter “The Campaign Courier” (Winter issue 2010) distributed to 1800 readers and the RSPCA Victoria volunteer e-newsletter (Winter issue 2010) distributed to 800 readers. Interested readers were directed to an online version of the survey using the commercial software (SurveyMonkey 1999–2013). The advertisement gave a brief outline (<140 words) of the research and a web link for the survey. Of the 6330 questionnaires distributed within New Zealand, 164 (2.6%) were returned. Within Australia 142 (1.5%) online questionnaire submissions were received. The nature of the questionnaire distribution in both New Zealand and Australia prevented us from determining the number of original questionnaires and advertisements that reached the nominated target audience (companion animal owners who had owned more than one companion animal with at least one of these animals having passed away in the previous five years). 

All aspects of this research were approved by the Unitec Ethics and Research Committee, Auckland, New Zealand (2010-1044) and the Behavioural & Social Sciences Ethical Review Committee, University of Queensland, Australia (2010000337).

### 2.3. Statistical Analysis

Data relevant to dogs and cats was analysed using Minitab version 16 (Minitab Inc., PA, USA). The data generally followed a non-parametric distribution pattern established using the Anderson Darling Test for normality, and hence non-parametric tests were engaged. Descriptive statistics were used to describe reported behavioural changes and their associated duration. Cross-tabulation with Chi-Squared (χ*^2^*) test of association (or Fisher’s Exact Test, where appropriate) was used to investigate associations between the species of deceased animal and the remaining animal. Significance was assumed if *p* < 0.05.

## 3. Results

### 3.1. Demographic Data

Of the total 306 surveys received, 37 were incomplete and therefore discarded from analysis. The resulting sample of respondents (*n* = 279) comprised 254 females and 23 males, with 6% of respondents aged 18–25, 11% 26–35, 21% 36–45, 31% 46–55, 19% 56–65 and 13% 66+ years. Seven hundred and seventy animals were represented, of which 356 were recently deceased (within the previous 5 years). Of the remaining (surviving) animals (*n* = 414), 75% (*n* = 311) were identified by their owner as showing at least one behavioural change (159 dogs, 152 cats) subsequent to their companion passing away. Animal demographic data are summarised in Table 1. 

### 3.2. Behavioural Changes

Following the loss of an animal companion, owners reported a mean of 4.8 ± 0.2 behaviour changes in their dogs and 4.5 ± 0.2 in their cats. 

Of the most commonly reported behavioural changes in dogs (*n*_tot_ = 153), 74% (*n* = 113) were reported to display changes in affectionate behaviour, of which 35% (*n* = 53) were described as more demanding of attention from their owner, 26% (*n* = 40) were described as behaving in a clingy/needy manner. By contrast, 10% (*n* = 16) were described as seeking less affection from their owner (Table 2). A total of 60% (94/159) of dogs were reported to display changes in territorial behaviour, of which 30% sought out the deceased’s favourite spot (Table 2). Changes in sleep behaviour were reported for 42% (67/159) of the dogs, with 34% displaying an increase in duration (Table 2). The amount and speed of food consumption was also reported to change in 42% (66/159) and 34% (55/159) of dogs respectively, with a decrease in volume observed in 35% and speed in 31% of the dogs (Table 2). Affectionate and sleep behaviour changes were reported to last for a median duration of 2–6 months, whilst changes in territorial and feeding behaviour were reported to last for a median duration of two months or less. 

For cats, the most commonly reported behavioural changes included variation in affectionate behaviour (78% of cats; 117/150), with 40% (*n* = 60) of cats reported to have demanded more affection from their owner, whilst 22% (*n* = 33) became increasingly clingy/needy and 15% (*n* = 22) sought less affection (Table 2). Sixty-three percent of cats were reported to display a change in territorial behaviour (96/152), of which 36% were reported to seek out the deceased animal’s favourite spot (Table 2). Additionally, the frequency of vocalisations were reported to change in 46% (70/152) of cats with 43% displaying an increase, whilst in 35% (53/152) of cats the volume of vocalisations was reported to change with 32% displaying an increased level (Table 2). For cats, the duration of changes in affectionate behaviour was reported to last for a median duration of between 2 and 6 months, whilst the median duration of changes in territorial and vocalisation behaviour was reported as 2 months or less. 

### 3.3. Species Influence

Eighty two percent (*n* = 339) of the remaining animals that had lost a companion dog or cat. For remaining dogs, 79% (*n* = 159) were reported to have lost a companion dog, whilst, 18% (*n* = 36) had lost a companion cat. For remaining cats, 59% (*n* = 125) had lost a companion cat and 9% (*n* = 19) a companion dog. Whether the remaining animal’s companion was of the same species was only found to have a statistical impact on one reported behaviour—the speed of food consumption. Remaining dogs who had lost a companion dog were more likely than those that had lost a companion cat to display a reduction in the speed with which they consumed food (χ*^2^* = 1.08, df = 1, *p* = 0.0046). No significant difference was found between the duration of time for which behaviour changes occurred and whether the lost companion animal was the same, or different, to that of the remaining animal.

### 3.4. Seeing the Deceased

One hundred and fifty-eight remaining animals (dogs 58%; cats 42%) viewed their deceased companion’s body. No difference was reported by owners between animals that saw, and those that did not see their companion’s deceased body on the reported behavioural changes (χ*^2^* = 1.825, df = 1, *p* = 0.22). Upon viewing the deceased animal, the majority (73%; *n* = 115) were reported to sniff and investigate the body of the dead companion.

## 4. Discussion

This study was a preliminary investigation into owner-perceived behavioural changes observed in companion animals following the loss of an animal companion. The most common behavioural change recognised and reported by owners was a change in the remaining animals’ affectionate behaviour. The majority of both dogs (74%) and cats (78%) displayed a change, with 82% of these dogs and 97% of cats demanding more affection from, and/or, becoming more clingy/needy towards the owner. This behaviour appears to parallel two well reported components of owner-orientated separation anxiety in dogs; the anxious maintenance of close proximity displayed towards owners upon the presentation of departure cues, and the over exuberant affection directed at the owner upon their return [52,53,54] and could reflect an inner emotional experience of grief, anxiety, or distress. Alternatively this behavioural change could result from reduced competition for access to the owner, or a reflection of the absence of social facilitation previously caused by the presence of a conspecific.

Another key behavioural indicator of owner-orientated separation anxiety in both dogs and cats is increased vocalisations [53,54,55,58,59,62,73]. Owners of cats in the present study reported changes in both frequency (46%) and volume (35%) of vocalisations with 93% and 92%, respectively, reported to increase, suggesting that the loss of a companion animal might be associated with the experience of anxiety. Aside from being a behaviour commonly associated with human-oriented separation anxiety, increased vocalisations following separation from conspecifics has also been documented in a range of farm animal species (goats, pigs, heifers, beef calves, lambs, dairy cows, horses [74,75,76,77,78,79,80]), a range of primate species [36,39,40,41,42,43,44,45] and elephants [30,81]. Increased vocalisations following the loss of an animal companion also parallel the first phase of the bi-phasic definition of grief which is characterised by increased searching activity and vocalisations [24]. 

Cats were also reported to show an increase in aggression directed towards other animals within the household. To our knowledge there is no literature on how cats behave as a result of the loss of a companion, aggressive behaviour is considered one of the most obvious signs of stress in cats [82]. Conversely, it is worth considering that an alternate explanation for the increased level of aggression reported in cats may have resulted from a change in hierarchical status resulting from the loss of a conspecific.

A large proportion of both dogs (60%) and cats (63%) were reported by their owners to display a change in territorial behaviour. More specifically, 50% of these dogs and 56% of these cats were described as seeking out the deceased animals’ favourite spot. Schultz et al. [51] reported that 27% of dogs and 41% of cats, subsequent to the loss of a conspecific, sought out the deceased animals’ favourite spot. Douglas-Hamilton et al. [30] have documented many elephant families travelling large distances to view the carcass of a deceased matriarch, and baboons have also been recorded searching for deceased infants when a troop re-enters an area where the infant died [33]. Consequently, this change in territorial behaviour may constitute the increased searching element of the first phase of the bi-phasic definition of grief. However, it is also possible that this behavioural change is the result of the remaining companion animal claiming this newly vacated territory as their own. Cats in particular are a very territorial species and the death of a companion animal could be perceived by the remaining animal as the removal of a resource competitor. Cats experiencing stress and decreased welfare tend to show increased hiding behaviour [83] and in the present study there were very low levels of this behaviour reported by owners (9%). Further research is required to test out these alternate theories.

In the case of dogs, owners reported a change in the amount of food consumed (42%) and speed with which the food was consumed (34%) after the loss of a companion animal. Eighty three percent of these dogs displayed a reduction in the amount of food consumed, whilst 91% displayed a reduction in speed. This reduction in both quantity and speed may be a negative effect of the loss of a companion animal. Dogs suffering from owner-oriented separation anxiety often abstain from eating and drinking [61] and recently cognitive bias testing has demonstrated that dogs suffering from owner-oriented separation anxiety show a decreased latency to approach a potential food reward [56]. Similar changes in the eating habits of both cats and dogs were reported by Schultz et al. [51], with both species showing a reduction in consumption following the death of a conspecific (36% of dogs and 46% of cats). In the case of remaining dogs a reduction in the speed of food consumption was increasingly likely to occur if the deceased companion animal was a dog, which might reflect a stronger bond between these particular conspecifics. Alternately, it might be an artefact of the removal of a resource competitor rather than a stress response resulting from the loss of a companion. 

Dogs (42%) were also reported to change their sleeping patterns. Changes in sleep patterns have been documented as a result of social isolation in rats [21], several species of primates [36,84] and farm animals [77], with a reduction in sleep being the most commonly reported change. In our study, 81% of the dogs reported to display a change in sleeping behaviour, were described as showing increasing levels of sleep which may parallel the second phase of the bi-phasic definition of grief which is characterised by decreased activity and withdrawal from the environment [24]. On the other hand increased sleep could be the result of less stimulation from a companion rather than an indicator of stress resulting from loss of an animal companion. To elucidate further on this finding, future research should obtain information regarding the duration of contact between the two companions and the nature of their cohabitation.

Our results show that witnessing the deceased animals’ bodies did not have a significant impact on behaviour. Behavioural changes were observed regardless of whether the deceased’s body was shown to the remaining animal. This might suggest that loss resulting from the removal of a companion animal from the household (e.g., sale or rehoming) could have a similar impact on behaviour, however further research is required to clarify this. Of the animals in the present study that were shown their companion’s deceased body, 73% sniffed and investigated the body. In other species, particularly primates and species considered to have high cognitive abilities (e.g., elephants and dolphins), behavioural interactions with conspecific carcasses have been reported. African elephants, for example, systematically investigate the bones and tusks of deceased elephants they encounter, visit the bones of relative elephants and display dramatic reactions towards dead and dying elephants [30,85]. Dolphins also show great interest in deceased individuals [31]. Japanese macaques have been documented to carry their deceased infants for up to a week before abandoning them [86]. Chimpanzees have been reported to carry their deceased infants for up to two months [87] before abandonment, and Cronin et al. [88] have described a behavioural response, including carrying and touching, of a chimpanzee mother towards her dead infant. Such interactive behaviour with the bodies of deceased animals has not just been reported as a result of the mother-offspring bond but also as a result of the severance of peer bonds. Brown [84] (p. 174) provides the following detailed behavioural response of an adult male chimpanzee to the loss of his mating partner. “*After the death of the female… the remaining one [male] made repeated attempts to arouse her, lifting up her head and hands, pushing her violently and rolling her over… The day following [removal of the body], he sat still most of the time and moaned continuously.*” 

### Limitations

Anthropomorphism may have played a role in owner descriptions of their animals’ behavioural changes. This is an extension of the human ability to empathise with another individual, amplified to the extent that we attribute human emotions and feelings to another species without scientific evidence that they are capable of such feelings. Thus the owner reports of behavioural changes suggestive of grief presented here may be a reflection of the grief that the owner is experiencing or a behavioural change that has occurred as a result of the change in owner behaviour resulting from their own feelings of grief. A large number of studies have shown that companion animal owners describe human-dog interactions more anthropomorphically than non-owners [89,90,91,92]. Additionally, the “human substitute” function that companion animals play is likely to encourage owners to believe that their animals can experience more complex emotional responses than would be attributed by non-owners. Conversely, the close-lived nature of companion animal–human relationships allows owners to understand animals in a variety of ways which go beyond the ideas of instinctual behaviour responses and potentially allow owners to recognise the individual subjectivity of an animal [93].

Furthermore, we must acknowledge a likely bias in our respondent sample group. We asked respondents to nominate themselves to participate in this research and it is very likely the owners that responded did so because they saw a change in their animals’ behaviour and because they remembered that change, whereas owners that did not see changes in behaviour may have been less likely to respond to our questionnaire. Given that our method of distribution was mainly through an animal welfare organisation, bias towards owners showing a greater motivation for animal behaviour and welfare issues may exist. Moreover, owners that believed companion animals capable of experiencing grief were likely more willing to respond to a questionnaire about animal grief. To address this limitation, future research could include a suite of questions about the owner’s belief in animal mind [94]. Additionally, the 5 year limit following the occurrence of an animal passing was set to ensure respondents described behavioural changes following recent deaths. It must be acknowledged that as increasing time passes the accuracy of memory decreases. Future research should aim to collect data as events occur from a carefully selected target population, however, the delicate nature of the topic makes identifying and approaching the target audience something that requires careful consideration and planning.

Despite these limitations there was consensus among respondents that the behaviour of companion animals changed as a result of the loss of a companion animal. These behavioural changes appear to parallel behaviours observed during owner-orientated separation related anxiety. Increased knowledge of the common behavioural changes experienced by remaining animals raises the possibility that these behaviours can be addressed and discussed within the companion animal industry. Future research should attempt to disentangle the influencing variables through direct assessment of animal behaviour following the removal of an animal companion, though the sensitive nature of the subject may constrain participation of companion animal owners.

## 5. Conclusions 

Companion animal owners reported behavioural changes in their animals following the loss of a companion. Further analysis of behavioural changes, independent of owner interpretation, is required to establish whether these behavioural changes are indeed a reflection of loss, result from a reduction in competition for resources or are a consequence of change in owner behaviour resulting from either their own feelings of grief or concern that their companion animal is grieving. 

## Figures and Tables

**Table 1:** Demographic information on deceased and remaining companion dogs and cats reported by respondents to a self-selected questionnaire.

	Dog			Cat	
	Deceased *n* (%)	Remaining *n* (%)		Deceased *n* (%)	Remaining *n* (%)
**Gender**					
Female	117 (59)	102 (52)		73 (47)	102 (52)
Male	82 (41)	96 (48)		83 (53)	94 (48)
Total	199	198		156	196
**Age**					
Juvenile	3 (2)	8 (4)		11 (7)	11 (6)
Adult	53 (29)	116 (59)		33 (22)	89 (46)
Senior	125 (69)	73 (37)		109 (71)	95 (49)
Total	181	197		153	195
**Breed**					
Toy	9 (5)	11 (6)	Pedigree	31	46 (24)
Terrier	25 (13)	36 (19)	Domestic	123	147 (76)
Gundog	29 (15)	28 (15)	Total	154	193
Hound	7 (4)	9 (5)			
Working	39 (20)	30 (16)			
Utility	19 (10)	18 (6)			
Non-Sporting	13 (7)	11 (6)			
Crossbreed	57 (29)	50 (26)			
Total	198	193			
**Cause of Death**					
Natural Causes	52 (27)			28 (18)	
Euthanasia	95 (49)			37 (24)	
Road Accident	10 (5)			35 (23)	
Other	37 (19)			52 (34)	
Total	194			152	
**Behaviour Directed Towards Deceased**					
Hissed or growled		0 (0)			4 (7)
Smelled and investigated		70 (78)			45 (74)
Showed no interest		12 (13)			11 (18)
Other		8 (9)			1 (2)
Total		90			61

**Table 2:** Number (%) of dogs and cats that displayed a behavioural change identified by their owners following the death of a companion animal.

Behaviour Category	Behaviour Sub Category	How the Behaviour Changed	Dogs (*n* = 159)	Cats (*n* = 152)
			Yes (%)	Yes (%)
Feeding	Amount of food consumption	Increased	9 (6)	14 (9)
		Decreased	55 (35)	32 (21)
		Ceased	2 (1)	4 (3)
		No change	93 (58)	102 (67)
	Speed of food consumption	Increased	5 (3)	3 (2)
		Decreased	50 (31)	18 (12)
		No change	104 (66)	131 (86)
	Did this change to feeding behaviour affect the animal’s health?	Yes	14 (9)	14 (9)
		No	144 (91)	137 (91)
Sleeping	Amount of time spent sleeping	Increased	54 (34)	30 (20)
		Decreased	13 (8)	7 (5)
		Ceased	0	0
		No Change	92 (58)	115 (76)
	Avoided regular sleeping spots	Yes	24 (15)	23 (15)
		No	135 (85)	129 (85)
Vocalisation	Amount of time spent vocalising	Increased	42 (27)	65 (43)
		Decreased	15 (10)	3 (2)
		Ceased	5 (3)	2 (1)
		No change	92 (60)	82 (54)
	Volume of vocalisations	Increased	30 (19)	49 (32)
		Decreased	12 (8)	4 (3)
		No change	117 (73)	99 (65)
Elimination	Occurrence of elimination/toileting	Increased	8 (40)	6 (46)
		Decreased	4 (20)	1 (8)
		No change	8 (40)	6 (46)
	Location of elimination/toileting	Changed	13 (8)	8 (5)
		No change	146 (92)	144 (95)
Aggression	Aggression directed towards people	Increased	8 (5)	6 (4)
		Decreased	0	2 (1)
		No change	151 (95)	144 (95)
	Aggression directed towards animals	Increased	9 (6)	19 (12)
		Decreased	4 (2)	0
		No change	145 (92)	133 (88)
Affection		Demanded more affection	53 (35)	60 (40)
		Sought less affection	16 (10)	22 (15)
		Became clingy/needy	40 (26)	33 (22)
		Avoided contact	4 (3)	2 (1)
		No change	40 (26)	33 (22)
Territory		Seeks out deceased’s favourite spots	47 (30)	54 (36)
		Avoids deceased’s territory	16 (10)	8 (5)
		Seeks higher ground than usual	9 (5)	20 (13)
		Spent increased time hiding	22 (14)	14 (9)
		No Change	65 (41)	56 (37)
